# The Neolithic Demographic Transition in Europe: Correlation with Juvenility Index Supports Interpretation of the Summed Calibrated Radiocarbon Date Probability Distribution (SCDPD) as a Valid Demographic Proxy

**DOI:** 10.1371/journal.pone.0105730

**Published:** 2014-08-25

**Authors:** Sean S. Downey, Emmy Bocaege, Tim Kerig, Kevan Edinborough, Stephen Shennan

**Affiliations:** 1 Department of Anthropology, University of Maryland, College Park, Maryland, United States of America; 2 Institute of Archaeology, University College London, London, United Kingdom; 3 Research Training Group 1878 “Archaeology of Pre-Modern Economies”, University of Cologne, Cologne, North-Rhine Westphalia, Germany; Bristol University, United Kingdom

## Abstract

Analysis of the proportion of immature skeletons recovered from European prehistoric cemeteries has shown that the transition to agriculture after 9000 BP triggered a long-term increase in human fertility. Here we compare the largest analysis of European cemeteries to date with an independent line of evidence, the summed calibrated date probability distribution of radiocarbon dates (SCDPD) from archaeological sites. Our cemetery reanalysis confirms increased growth rates after the introduction of agriculture; the radiocarbon analysis also shows this pattern, and a significant correlation between both lines of evidence confirms the demographic validity of SCDPDs. We analyze the areal extent of Neolithic enclosures and demographic data from ethnographically known farming and foraging societies and we estimate differences in population levels at individual sites. We find little effect on the overall shape and precision of the SCDPD and we observe a small increase in the correlation with the cemetery trends. The SCDPD analysis supports the hypothesis that the transition to agriculture dramatically increased demographic growth, but it was followed within centuries by a general pattern of collapse even after accounting for higher settlement densities during the Neolithic. The study supports the unique contribution of SCDPDs as a valid demographic proxy for the demographic patterns associated with early agriculture.

## Introduction

The transition from foraging to farming economies resulted in the Neolithic Demographic Transition (NDT) [1], which enabled higher population levels worldwide linked to a new regime of high fertility and mortality rates. One important source of evidence on palaeodemography is the analysis of human skeletal remains, and a variety of indices designed to estimate the proportion of juvenile skeletons within populations have provided unique insights into population growth rates and the structure of prehistoric human populations (cf. [2], [3]). These metrics have been widely used to analyze the timing and effects of early agriculture on the structure of human populations [4]–[6], and the observation of a higher proportion of juveniles after the introduction of farming has supported claims that fertility surged following the introduction of agriculture in Europe and other parts of the world [1]. Such insights helped refute the population pressure model, a long-standing claim by Binford [7] and others that demographic growth during the Mesolithic preceded and drove the agricultural transition [8].

However, the paleodemographic approach using skeletal remains and juvenility indices depends on accurate determination of age-at-death and it can be confounded by small sample sizes and under-representation of younger age-classes [8], [9]; moreover, cemeteries may accrete over long periods so they cannot usually be given a single precise date. A complementary method for assessing prehistoric population levels has been developed based on the distribution in time and space of radiocarbon dates [10], [11]. A recent analysis has detected a statistically significant boom-and-bust pattern following the introduction of agriculture in multiple sub-regions across Europe, and the absence of correlation with paleoclimate [12]. One advantage of this approach is that radiocarbon dates are substantially more abundant than skeletal remains; however, questions remain regarding the demographic relevance of SCDPDs because they are an indirect proxy for demographic levels. In particular, it is important to account for the fact that forager settlements would likely have been smaller than farmer settlements, since the SCDPD method normally assigns equal demographic weight to each archaeological period. Here, a direct comparison and statistical correlation of the growth rates inferred with the juvenility index and the relative population levels inferred with the SCDPD approach is undertaken. We also analyze the effects of farmer and forager settlement sizes, in order to validate the demographic patterns indicated by the SCDPDs and to evaluate the relative precision of each method.

## Methods and Materials

**Figure 1:**
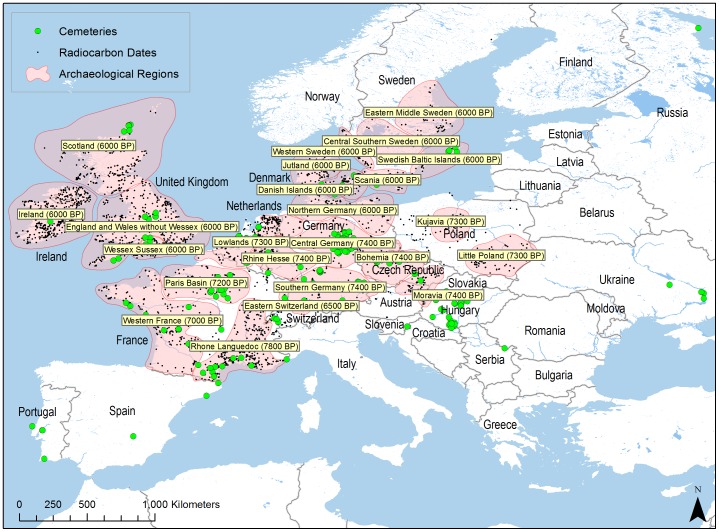
Study area indicating the location of cemeteries and anthropogenic radiocarbon dates. Early agriculture dates are shown for twenty-four well-documented archaeological regions: Southern Germany (n = 391), Bohemia (121), Central Germany (359), Central Southern Sweden (107), Danish Islands (298), Eastern Middle Sweden (101), Eastern Switzerland (275), England and Wales without Wessex (1188), Ireland (1721), Jutland (384), Kujavia (460), Little Poland (369), Lowlands (763), Moravia (287), Northern Germany (676), Paris Basin (571), Rhine Hesse (308), Rhone/Languedoc (978), Scania (234), Scotland (579), Swedish Baltic Islands (84), Wessex Sussex (581), Western France (494), Western Sweden (111). Map created using ArcGIS 10.0 by ESRI. Map data sources: ESRI, ArcWorld, NASA, NGA, DCW, USGS, EROS, and JRC CCM.

Here we analyze two independent lines of evidence: age-distributions of skeletons in European cemeteries [1], and a large number of radiocarbon dates collected by the EUROEVOL research team that will be made publically available in 2015 (http://www.ucl.ac.uk/euroevol). The study area spans Central and Northwestern Europe and covers 8,000–4,000 Cal. BP (Fig. 1). All data were collected from published sources, so no new field studies or specific permissions were required. The agricultural practices that triggered the NDT began in southwest Asia and moved west across Europe over 4,000 years. Following Bocquet-Appel [3] a relative chronology was used to analyze cemetery and radiocarbon data for regular patterns of demographic change using a zero point at the local arrival date of agriculture and a time scale in terms of years before and after the local arrival date. All statistical procedures were written in R [13].

### Cemetery Analysis

To maximize comparability, our cemetery dataset included the 67 used in Bocquet-Appel [14], and additional cemeteries we identified in other published sources to bring the total to 212. The criterion for including cemeteries in the dataset was when publications included age-class information, reliable date estimates, evidence for cultural homogeneity, and evidence for “natural” as opposed to “violent” death [3]. We omitted sites with poor dating accuracy, other cemeteries with minimum number of individuals (MNI) less than 10, and we pooled contemporaneous burials from local regions when contexts were secure. 101 cemeteries met these criteria (Table S1). The binomial proportion *P(5–19)* was calculated as the proportion of skeletons aged 5–19 within cemeteries after omitting individuals aged 0–4 [3]. P(5–19) is referred to as the *juvenility index* throughout this paper. In cases where age classes were ambiguous in the original publications, the risk of mortality was evenly distributed across corresponding age-classes (following CA+ Appendix, Rule 6 in [3]). Absolute cemetery chronologies and the date of the earliest local evidence of agriculture were estimated on a site-by-site basis from available archaeological information and a relative date was calculated as the difference between the two (DT).

To determine whether the boom/bust signal identified in [12] can be detected using cemetery data, the juvenility index was estimated for 10-year increments using a loess model (degree = 2, α = 0.40), and weighted by sample size. Alpha parameters were chosen manually for both the cemetery analysis and the radiocarbon analysis (described below) to minimize the chance of over-fitting. Next, long-term growth was modeled as a function of DT using a generalized linear model (GLM) with Gaussian error, also weighted by sample size. The Mesolithic and Neolithic periods were modeled separately, and significant departures of the loess model from the GLM indicate deviation from long-term growth trends. 95% confidence intervals were calculated because of the overall statistical weakness of the cemetery dataset, but to account for a multiple-testing bias due to the non-independence of each 10-year period under the loess model, a family-wise error rate (FWER) was also estimated with the Šidák correction and used to calculate corrected confidence intervals [15].

### Settlement Size Analysis

**Figure 2:**
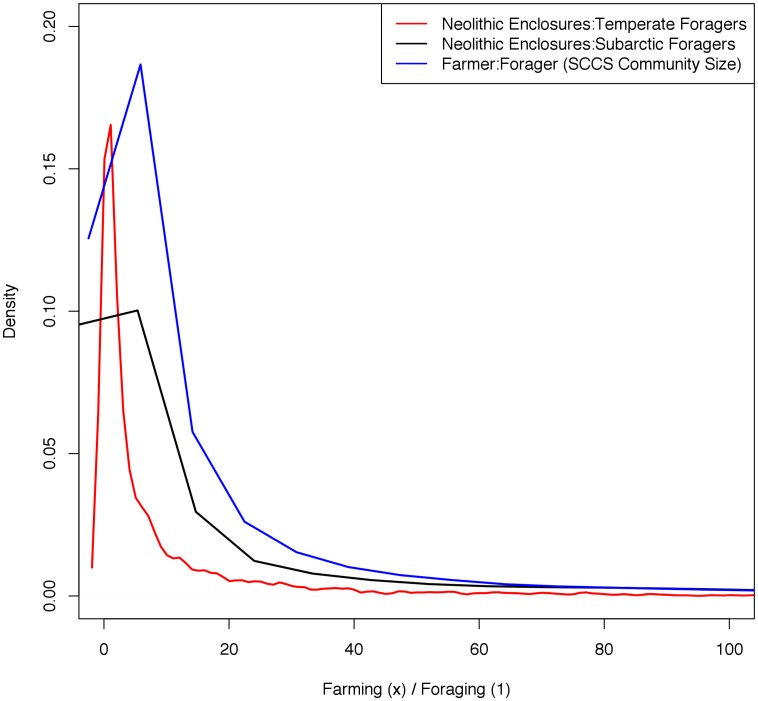
Distribution of bootstrapped farmer:forager settlement size ratios using ethnographic data from temperate and subarctic foraging groups, estimated sizes for Neolithic enclosures from archaeological excavations, and community size information from the SCCS.

The SCDPD method normally assumes that all site phases have equal demographic weight. However, it is likely that forager groups in terrestrial temperate European conditions would have been smaller on average than subsequent farming communities, so in order to make valid demographic comparisons this needs to be taken into account. While Neolithic settlement sizes are relatively easy to obtain, this is generally not the case for the Mesolithic because of the nature of occupation and the formation processes affecting their survival and discovery [16], [17]. Therefore, to obtain relevant scaling factors we first analyzed data from ethnographically known farming and foraging societies. Using the 186 ethnographically observed societies in Murdock and White's Standard Cross-Cultural Sample (SCCS) [18] we analyzed the code “Community Size” for each society, which allows direct assessment of a population ratio for foraging (n = 20) versus farming (n = 72) settlements (Fig. S1). We then performed a bootstrap analysis in which pairs of data points were sampled from the forager and farmer data (n = 100,000) and the distribution of population size scaling factors was calculated. The SCCS aggregates data using ordinal variables, so in order to generate a density distribution, the bootstrapping routine converted the ordinal variables with a uniform distribution encompassing the range of each ordinal level. The median farmer to forager population ratio is 3.66 (Table 1) and a strong right-skew can be detected in the distributions (Fig. 2).

**Table 1:** Results of bootstrap analysis comparing farming communities to foraging communities based on population counts and settlement size.

Site Size	Min	1Q	Median	3Q	Max
Enclosures: Temperate	0.00	0.53	2.46	10.06	507.3
Enclosures: Subarctic	0.00	0.25	2.14	19.39	4757.00
**Community Size**	**Min**	**1Q**	**Median**	**3Q**	**Max**
SCCS Farmers: Foragers	0.00	1.12	3.66	13.40	4551

The distribution is right-skewed therefore the median is the appropriate measurement of central tendency.

Numerous studies have shown a strong relationship between settlement area and population size (see [19] for a recent example that offers a theoretically-based explanation for the relationship). We were also able to compare the areas of settlements for a sample of temperate and sub-Arctic forager groups collected by Whitelaw [20] with the sizes of a sample of occupied Neolithic enclosures representing bounded settlement areas in our study area. The distribution of the site size data is shown in Figure S2. The fact that the temperate data include sedentary Northwest coast and California foragers while the sub-Arctic category represents mobile boreal forest groups makes this an appropriate comparison for the types of occupation to be found in Mesolithic Europe. We classified each source of data as either “farming” or “foraging” and compared settlement-size information from both forager groups to the Neolithic enclosure area measurements. We then performed a bootstrap analysis, as above, in which pairs of data points were sampled from the enclosures and foraging data and settlement area factors were calculated (n = 100,000), resulting in a median ratio of farmer to forager settlement size of 2.46 for temperate foragers and 2.14 for subarctic foragers (Fig. 2; Table 1).

In addition, empirical population levels and settlement extent estimates were also collected and summarized and we compared farming population and settlement areas to those for foragers to determine a further range of scaling factors representing the differences in areal extent and density between farming and foraging settlements (Table 2). Considering these ethnographic and archaeological sources pertaining to farmer and forager demography and settlement areas, and the bootstrap analyses described above, we estimate the overall population increase that occurred after the introduction of agriculture ranges between 2–8 farmers for every forager represented by each settlement. Therefore, a uniform distribution with this range is used to model the distribution of scaling factors for the SCDPDs based on site classifications in the original radiocarbon reports as either Mesolithic or Neolithic.

**Table 2:** Example ethnographic and archaeological settlement area and density data from published sources.

Economic system	Source	Area (ha)	Area Ratio	Pop.	Pop. Ratio	Settlement Density (per/ha)
Foraging	Subarctic & Temperate [20]	0.41 ++	-	40++	-	156.6
Farming	Neolithic Enclosures	1.3++	3.2∶1	**	-	-
Farming	Kur River Basin, Iran [38]	2.0+	4.9∶1	305+	7.6∶1	152
Farming	Central Highlands, Peru 1540 [39]	2.0++	4.9∶1	216(36)*++	5.4∶1	108

* Values within parentheses were reported as “tribute payers,” probably in reference to the male household head. A rough settlement population was estimated at 6 persons per household.

** Pop levels are unknown for Neolithic enclosures.

+ Indicates arithmetic mean reported in original publication.

++ Indicates median.

### Radiocarbon Analysis

For the radiocarbon analysis 8,023 dates from twenty-four regions with known European Neolithic populations were compiled into a database (See [11] for sources; Fig. 1). Uncalibrated Neolithic dates earlier than 500 years before the local arrival of agriculture were omitted from the analysis to reduce the earlier skewing that can result when old wood was recycled in prehistoric times for use during later occupation phases [21]. Dates were calibrated using MCMC simulation [22] and the IntCal09 radiocarbon date calibration curve [23]. Simulations were burned-in for 500,000 iterations and samples taken every 1,000 steps. The calibration process combined the observed dates with laboratory error and error from the IntCal09 calibration curve in a simulation that generated a posterior distribution of 11.4m dates incorporating calibration curve and lab error.

Over-dating of single site phases with respect to prehistoric population levels can occur at, for example, highly visible ceremonial sites. This was corrected by classifying dates into discrete occupation phases lasting no longer than 200 years and post-processing the posterior distribution so that only a single date occurred within each site-phase. This effectively distributed the observed variance within site-phases (n = 4,090) throughout the posterior distribution without overweighting site-phases that had more than a single date [11]. The next step was to convert the posterior date distributions from all regions into standardized DT time. An approximate agriculture date was estimated for each region from available archaeological information and DT was calculated as the difference between each calibrated date and its regional agriculture date. Roughly speaking, the agriculture dates for the more eastern and southern regions are c.7500–7200 cal. BP, while in northwest Europe they are c.6000 cal. BP.

To take into account the uncertainty in relative population estimates in the Neolithic distribution from the settlement size analysis, we calculated separate SCDPDs for the Mesolithic and Neolithic periods. For each 10-year bin in the Neolithic SCDPD we summed a sample from the uniform distribution of forager to farmer population ratios (2–8) equal in size to the frequency within the bin. The same Neolithic scaling function was applied when calculating confidence intervals (described below). The ‘scaled’ Neolithic SCDPD, the ‘un-scaled’ Neolithic SCDPD, and the Mesolithic SCDPD were analyzed separately, and also combined to calculate overall SCDPDs. For each of these samples, inferred demographic histories were modeled by estimating discrete summed calibrated radiocarbon date densities for each 10-year increment using a loess model (degree = 2, α = 0.15). A smaller α value (the proportion of adjacent points used in each local regression) was used to model the radiocarbon data than the value used in the cemetery model because larger sample sizes allowed for smaller values with less risk of over-fitting.

Next, long-term patterns of change in the SCDPDs were tested for significance. SCDPD population growth rates during the Mesolithic and Neolithic periods were modeled separately as a function of DT using a GLM with quasi Poisson-distributed error. Then, to generate confidence intervals, a sample of dates equivalent in size to the number of unique occupation phases was taken from the posterior distribution to correct for the possibility of over-dating, summed using 10-year bins, and a loess model was estimated (degree = 2, α = 0.15). This process was then repeated 10,000 times, such that each loess model effectively constitutes one “possible” demographic history, given known sources of error, including the uncertainty in relative population estimates. Uncorrected and Šidák-corrected confidence intervals were calculated because, as above, of the non-independence of the 10-year bins. Significant deviations between the confidence intervals and the GLM indicate periods of departure from long-term population trends (booms and busts). Demographic cycle durations were determined using the population maximum year, defined by the SCDPD maximum within 1000 years of DT = 0, and the minimum year by the lowest value 2,000 years thereafter.

### Cross-Correlating the Juvenility Index and SCDPDs

To investigate whether the overall SCDPD pattern is a valid demographic proxy, we calculated Pearson's product-moment correlation coefficients for the scaled and un-scaled combined Mesolithic and Neolithic SCDPD loess models, and the juvenility index loess model.

## Results

**Figure 3:**
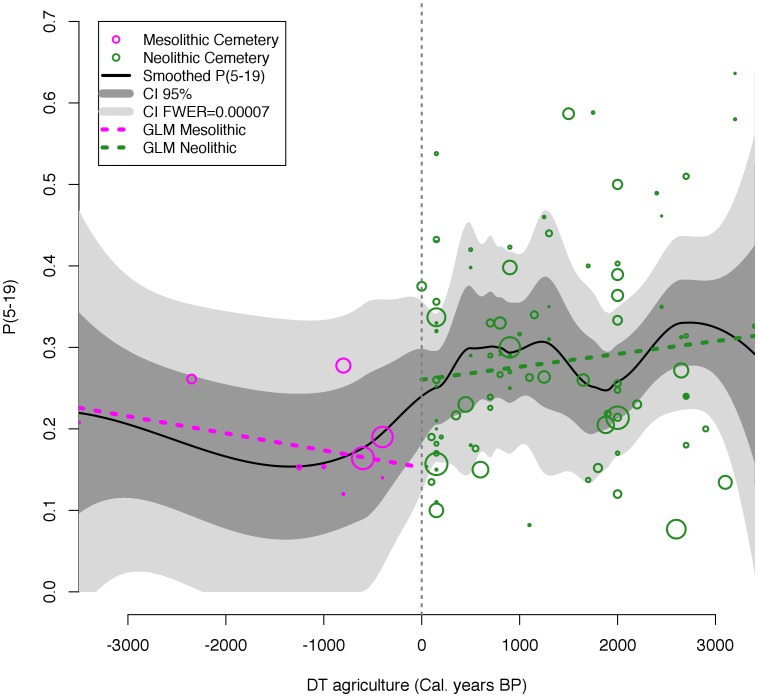
Analysis of the cemetery composition of immature skeletons using the juvenility index from 101 European cemeteries. Cemetery dates are adjusted for the beginning of agriculture at DT = 0 and negative DT values indicate Mesolithic populations. Confidence intervals indicate uncertainty due to sampling in the cemetery data. Point size indicates the minimum number of individuals (MNI) excavated from each cemetery.

During the Mesolithic, long-term trends in the juvenility index provide no evidence for any pattern other than a stationary population (Fig. 3; est.  = −2.094e-05, std. err. = 1.131e-05, t = −1.852, p = 0.1065). This supports the work of Jackes and others [24]. The first evidence of agriculture (DT = 0) signals a transition from Mesolithic foraging to Neolithic cultivation and a rising proportion of immature skeletons indicates a period of growth lasting around 720 years. This is followed by nearly 1,000 years of relative stability before an apparent decrease caused by higher occurrences of cemeteries with lower juvenility indices around DT 2000. This dip bears a striking resemblance to a “bust” phase, but the feature is not statistically significant because it appears entirely within the confidence intervals of the estimates. Neither is the long-term pattern of growth during the Neolithic significant (est. = 1.577e-05, std. err. = 1.172e-05, t = 1.345, p = 0.182). The difference between the median Mesolithic juvenility levels (M = 0.1641) and Neolithic levels (M = 0.2683) was found to be significant with a Wilcoxon rank sum test (W = 184, p = 0.0061); however, measurement of growth rates using the juvenility index is clearly limited by the paucity of the skeletal data, especially during the late Mesolithic/pre-Neolithic phases.

**Figure 4:**
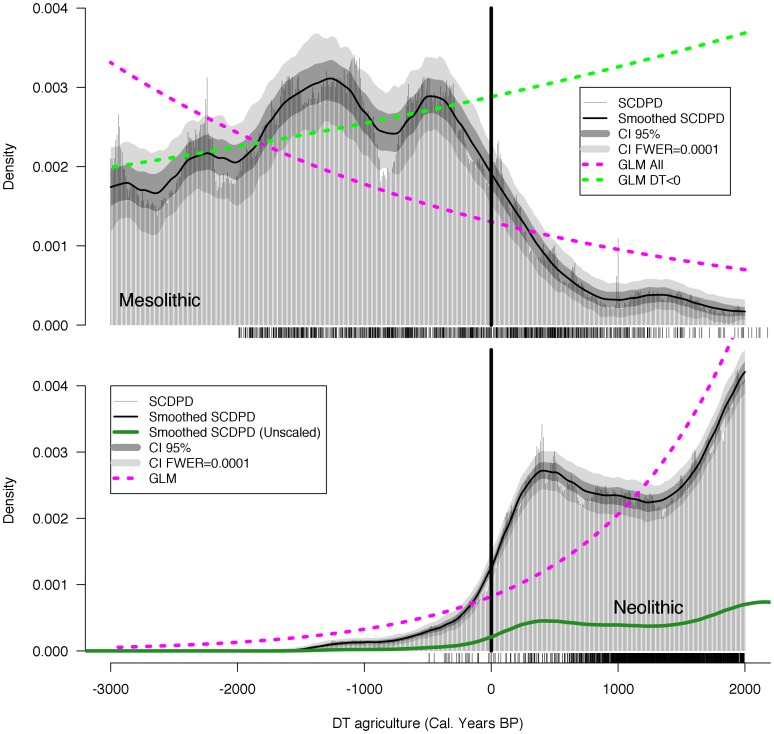
Analysis of the summed calibrated radiocarbon date density (SCDPD) curves for (A) Mesolithic and (B) Neolithic populations. All radiocarbon dates are adjusted for the beginning of agriculture at DT = 0 and negative DT values indicate Mesolithic populations. Confidence intervals indicate error introduced by sampling, variable atmospheric ^14^C accumulation rates and lab error, and differences in settlement size due to larger Neolithic populations. Each tick indicates one uncalibrated radiocarbon date.

**Figure 5:**
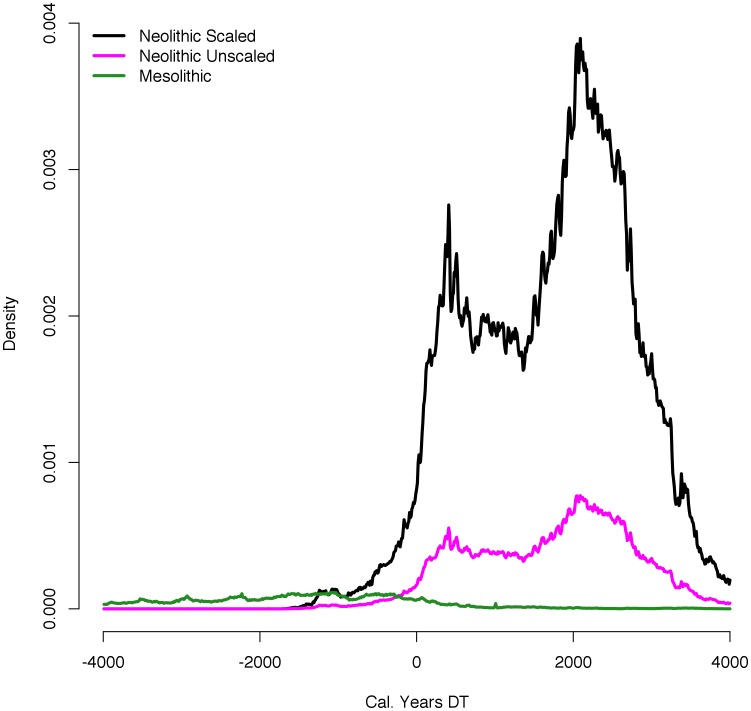
Comparison of Mesolithic SCDPD with scaled and unscaled Neolithic SCDPDs. The scaled Neolithic curve indicates the likelihood that Neolithic farming settlements had higher populations than Mesolithic foragers.

Summed radiocarbon date densities are a less direct proxy for population *structure* than the proportion of juvenile skeletons in cemeteries. However, radiocarbon dates are more prevalent than skeletons and in sufficient quantities may provide a more precise proxy for relative population *levels*. Fig. 4 shows the SCDPD and demographic growth models for the Mesolithic and Neolithic periods. During the Mesolithic (Fig. 4a) there is a clear decline in the SCDPD following the introduction of agriculture and two statistically significant GLMs are plotted: one including only Mesolithic sites predating agriculture (DT<0) that indicates Mesolithic populations were increasing slowly (est. = 1.232e-04, std. err. = 1.303e-05, t = 9.459, p <2e-16); and the second for the entire sequence that indicates that a dramatic decline in Mesolithic populations followed agriculture (est. = −3.110e-04, std. err. = 1.647e-05, t = −18.88, p <2e-16). Deviations between the corrected confidence intervals and the pre-agriculture GLM (DT<0) are detected around −1400 DT, hinting at Mesolithic population fluctuations. During the Neolithic period (Fig. 4b) there is a statistically significant pattern of growth (est. = 9.194e-04, std. err. = 2.412e-05, t = 38.11, p = <2e-16) and substantial deviations from the confidence intervals that indicate significant boom-bust fluctuations are detected. We note that the y-axes in Figure 4 use different scales because the absolute size of farming populations would have been dramatically higher during the Neolithic, as discussed above. This is illustrated in Fig. 5, where the effect of differential settlement sizes on the overall population pattern can be seen when the Mesolithic and Neolithic SCDPDs are plotted together. As Fig. 4b makes clear, the increase in Neolithic date densities prior to DT = 0 results from the inclusion of dates that have a culturally Neolithic context but are earlier than the estimated date for the local arrival of farming because of “old-wood” effects.

Cross-correlating the long-term population trends yielded a significant Pearson's estimate of the product-moment correlation coefficient for the un-scaled SCDPD (r = 0.855, lag = 0), and an even higher estimate for the scaled SCDPD (r = 0.883, lag = 0). This result validates the overall accuracy of the SCDPDs for inferring long-term population patterns.

The increased resolution provided by the radiocarbon proxy should allow more precise estimation of overall intrinsic growth rates during the early Neolithic. After the introduction of agriculture a 420-year period of growth was followed by approximately 840 years of decreasing population levels, yielding a complete demographic cycle lasting around 1260 years. The onset of population growth and collapse in the Neolithic is quite rapid, with intrinsic growth rates averaging 0.172% and −0.024% per year, respectively.

## Discussion

As noted above, the study area covered by the cemetery and radiocarbon data brings together two main agricultural expansions that occurred at different times. The first is the LBK expansion across Central Europe to the Paris Basin c.7500–7200 cal.BP, the second is the expansion into Britain, Ireland and southern Scandinavia over a millennium later, at c.6000 cal.BP. Bocquet–Appel [12] emphasizes that the effects on prehistoric demography cannot be properly assessed without controlling for these differences in the timing of the arrival of agriculture using DT time.

Our analysis of the demographic patterns using the juvenility index supports other recent work indicating that Mesolithic populations were stationary, neither growing or declining significantly over the long term [24]. However, rigorous statistical testing of the significance of the cemetery data indicates wide confidence intervals that may obscure important patterns. The non-significant dip beginning around DT 1200 years may be a statistical artifact of the cemetery analysis, but the SCDPD approach provides much narrower confidence intervals and reveals a statistically significant period of demographic collapse that can be timed and dated. Several paleodemographic studies using the juvenility index indicate a similar demographic increase following agriculture [3], [14], [25]–[29], and some indicate Neolithic declines. However, these features were explained simply as a “two-phase cycle” [3], a second increase due to increasing social complexity [29], or the decline feature simply appeared in a figure with little accompanying commentary.

As the results show, there is a strong correlation between the temporal trends in the juvenility index and in the SCDPDs, but the confidence intervals associated with the SCDPDs are narrower, indicating significant deviations from expected growth rates, and the Neolithic collapse appears clearly in the summed radiocarbon date densities (Fig. 3). The relative metabolic load hypothesis suggests that the energetic stress balances of the mother determine the effects of variation in lactation on fertility rates [14], [30], [31]. So the observed increase in population levels was probably due to changes in female energy balances produced by sedentism and the increased availability of carbohydrates associated with farming. However, research by Shennan et al. [12] indicates that the period of growth following the introduction of agriculture was followed by collapse in several regions across Europe. The authors found very little correlation to Holocene climate proxies and therefore suggested that climate change cannot be invoked as the primary driver of collapse at large spatial and temporal scales across Europe. They suggest that as agriculture and herding spread, local populations experienced long periods of growth, followed by comparable periods of demographic decline, and our results support this conclusion. The pattern repeated itself at different times and places, but it was regular and organized around the arrival of the agricultural ‘wave of advance’ [32].

One way to explain this pattern is by examining the duration of the boom-bust cycle as measured in DT time. Statistically significant departures from long-term growth rates in the Neolithic period, and perhaps also during the Mesolithic, suggest the presence of demographic cycling and we note that the durations reported above are on the upper end, but of the same order of magnitude, as a range of 120–450+ years predicted using an ecological predator-prey model parameterized for human life-cycles and then empirically estimated for historical and archaeological populations in Europe and Asia [33].

## Conclusion

This analysis of population age structure using cemetery assemblages and of relative population densities from SCDPDs both show that the introduction of farming was associated with major population growth. Analysis of archaeological and ethnographic data indicates between a two- and eight-fold difference in site population levels between foragers and farmers. The methods developed here apply this scaling factor as an additional source of uncertainty, in addition to non-linearity in the radiocarbon date calibration curve and lab error. The results are robust to the higher density of farmer settlements. The correlation of the SCDPDs with the juvenility index confirms SCDPDs as valid proxies of demographic processes, based as they are on different theoretical and methodological foundations. Thus we conclude that insomuch as early farming dramatically increased absolute population levels during the Early Neolithic (Fig. 5), it was a demographic success; however, it also appears to have induced dramatic population fluctuations, perhaps by amplifying preexisting population fluctuations, and resulted in significant demographic instability.

The discovery of consistent patterns of demographic collapse across Europe is a striking revision to our understanding of the consequences of the agricultural revolution. As mortality caught up with fertility under the new agricultural conditions it did not simply lead to stabilization of population levels following a smooth logistic curve. Declines were catastrophic, lowering population by 20.1% on average over 840 years and in some regions, for example Britain [11], approaching the rate of 30–60% during the Black Death [34]. Empirical evidence for collapse has previously been invisible in paleodemography due to the granularity of cemetery data and substantial uncertainty involved with calculating the juvenility index ratio over four millennia. In contrast, radiocarbon dates are abundant, the population reconstructions are more precise, and therefore statistical analysis can be more rigorous. Furthermore, at least over the short to medium term, there are unlikely to be much greater numbers of newly excavated and analyzed cemeteries, but radiocarbon date availability can be increased by carefully specified research designs.

The Neolithic launched an experiment in the dynamics of food production and demographic growth that is still under way. Here we have compared European cemetery data to summed calibrated radiocarbon date distributions in the most rigorous analysis of its kind. Our results demonstrate that agriculture, probably coupled with underlying demographic cycles, triggered significant instability, feedback, and precipitous population collapse. In the current demographic transition, where decreases in fertility are now catching up with falling mortality, but all too slowly [35], [36], there is also no guarantee of a smooth logistical leveling out of world population levels. In contemplating paths to a sustainable future for humanity, it is worth considering the consequences of failure during the transition from foraging to farming in Europe between eight and six thousand years ago [37].

## Supporting Information

Figure S1 Community Size Distribution from SCCS.
(DOC)

Figure S2 Comparison of the distribution of area (ha) for Neolithic enclosures, sub-arctic forager settlements, and temperate foraging settlements.
(DOC)

Table S1 Mesolithic and Neolithic cemeteries used in the analysis.
(DOC)
