# Supplementary material for: The Neolithic Demographic Transition in Europe: Correlation with Juvenility Index Supports Interpretation of the Summed Calibrated Radiocarbon Date Probability Distribution (SCDPD) as a Valid Demographic Proxy
Source: PLoS One. 2014 Aug 25;9(8):e105730. doi: 10.1371/journal.pone.0105730 (PMC4143272; doi:10.1371/journal.pone.0105730)
Supplement: Figure S1 — Community Size Distribution from SCCS. (DOC) [file pone.0105730.s001.doc]

Supplemental Figure S1. Community Size Distribution from Standard Cross Cultural Sample (SCCS). Farming communities were included when the value “Primarily agricultural” was indicated for the variable, “3. AGRICULTURE- CONTRIBUTION TO LOCAL FOOD SUPPLY”. Foraging communities were identified when any of the following variables were listed as “> 50%”: “7. FISHING- CONTRIBUTION TO FOOD SUPPLY”, “ 9. HUNTING- CONTRIBUTION TO FOOD SUPPLY”, and “11. GATHERING- CONTRIBUTION TO FOOD SUPPLY.” Only communities with fewer than 5,000 individuals were included.
