# Supplementary material for: The Neolithic Demographic Transition in Europe: Correlation with Juvenility Index Supports Interpretation of the Summed Calibrated Radiocarbon Date Probability Distribution (SCDPD) as a Valid Demographic Proxy
Source: PLoS One. 2014 Aug 25;9(8):e105730. doi: 10.1371/journal.pone.0105730 (PMC4143272; doi:10.1371/journal.pone.0105730)
Supplement: Figure S2 — Comparison of the distribution of area (ha) for Neolithic enclosures, sub-arctic forager settlements, and temperate foraging settlements. (DOC) [file pone.0105730.s002.doc]

Supplemental Figure S2. Comparison of the distribution of area (ha) for Neolithic enclosures, Sub-arctic forager settlements, and temperate foraging settlements. All ethnographic foragers settlement size measurements are taken from Appendix 1 and Appendix 2 of (Whitelaw 1989; see ref. [20] in main text). Data for temperate and subarctic groups were selected using the following criteria: *Data.reliability* (1-3), *Completeness.of.plan* (1-4), *Social.unit.represented* (1-5), *Traditional.character* (1-3), *Economic.orientation* (1-3), and *Duration.of.occupation* (1-6).
