# Supplementary material for: The Neolithic Demographic Transition in Europe: Correlation with Juvenility Index Supports Interpretation of the Summed Calibrated Radiocarbon Date Probability Distribution (SCDPD) as a Valid Demographic Proxy
Source: PLoS One. 2014 Aug 25;9(8):e105730. doi: 10.1371/journal.pone.0105730 (PMC4143272; doi:10.1371/journal.pone.0105730)
Supplement: Table S1 — Mesolithic and Neolithic cemeteries used in the analysis. (DOC) [file pone.0105730.s003.doc]

Supplemental Table S1. Mesolithic and Neolithic cemeteries used in the analysis.

| **Mesolithic Sites** | | | | | | | | | | | | | |
| --- | --- | --- | --- | --- | --- | --- | --- | --- | --- | --- | --- | --- | --- |
| **No.** | **Site name** | **Source** | **Country** | **Front (BP)** | **Mean date (BP)** | **dt** | **0.4** | **5.19** | **20** | **child** | **unk** | **MNI** | **ratio** |
| **1** | **Hardinxveld-Giessendam ‘Polderweg’ and De Bruin** | [1] | Netherlands | 6300 | 8300 | -800 | 0.6 | 2.2 | 16.2 |  |  | 19 | 0.12 |
| **2** | **Hoëdic[[1]](#footnote-2)** | [1-4] | France | 6800 | 8800 | -400 | 3 |  | 9 | 2 |  | 14 | 0.19 |
| **3** | **Teviec[[2]](#footnote-3)** | [1,3-5] | France | 6800 | 8800 | -400 | 6 |  | 15 | 2 |  | 23 | 0.14 |
| **4** | **Columnata[[3]](#footnote-4)** | [6] | Algeria | 8500 | 10500 | -2350 | 50.37 | 16.62 | 47 |  |  | 114 | 0.2612 |
| **5** | **Đerdap** | [6,7] | Serbia | 8000 | 10000 | -600 | 53 | 22 | 112 |  |  | 187 | 0.1641 |
| **6** | **Moita do Sebastiao** | [6,8,9] | Portugal | 7500 | 9500 | -1251 | 22.7 | 17.33 | 96 |  |  | 136 | 0.1529 |
| **7** | **Skateholm** | [10] | Sweden | 6000 | 8000 | -1000 | 6 | 8 | 44 |  |  | 58 | 0.1538 |
| **8** | **Taforalt (Grotte des Pigeons)** | [6] | Morocco | 7500 | 9500 | -3500 | 78 | 21 | 80 |  |  | 179 | 0.2079 |
| **9** | **Vedbaek** | [6,11] | Denmark | 6000 | 8000 | -800 | 5 | 5 | 13 |  |  | 23 | 0.2777 |
| **10** | **Ascott-under-Wychwood** | [12,13] | UK | 5900 | 7900 | 150 | 1 | 3 | 17 |  |  | 21 | 0.15 |

| **Neolithic Sites** | | | | | | | | | | | | | |
| --- | --- | --- | --- | --- | --- | --- | --- | --- | --- | --- | --- | --- | --- |
| **No.** | **Site name** | **Source** | **Country** | **Front (BP)** | **Mean date (BP)** | **dt** | **0.4** | **5.19** | **20** | **child** | **unk** | **MNI** | **ratio** |
| **11** | **Aszod-Papiföldek** | [14,15] | Hungary | 7600 | 9600 | 900 |  |  | 124 | 78 | 2 | 204 | 0.3 |
| **12** | **Hungary, Budapest, Baden culture (Balatonmagyarod-Hidvegpuszta-Deli, Budapest-Andor, Budapest-Bekasmegyer, Budapest-Kaposztasmegyer, Palotabozsok, Pecs-Hoeromu, Pecs-Vasas-Homokbanya)** | [15] | Hungary | 7600 | 9600 | 2700 | 0 | 5 | 16 |  |  | 21 | 0.24 |
| **13** | **Čachovice[[4]](#footnote-5)** | [16] | Czech republic | 7400 | 9400 | 2700 |  |  | 39 | 12 |  | 51 | 0.24 |
| **14** | **Carsington Pasture Cave** | [17] | UK | 5800 | 7800 | 1300 |  |  | 9 | 11 |  | 20 | 0.31 |
| **15** | **Collombey-Muraz, Barmaz[[5]](#footnote-6)** | [18,19] | Switzerland | 7250 | 9250 | 1150 |  |  | 42 | 28 |  | 70 | 0.34 |
| **16** | **Corseaux-sur-Vevey, en Seyton** | [18,20] | Switzerland | 7250 | 9250 | 1650 | 7 | 7 | 28 |  | 9 | 51 | 0.26 |
| **17** | **Csabdi-Telizoldes** | [15] | Hungary | 7600 | 9600 | 900 | 0 | 3 | 9 |  |  | 12 | 0.25 |
| **18** | **Fussel's lodge** | [21] | UK | 5900 | 7900 | 100 |  |  | 26 | 8 |  | 34 | 0.19 |
| **19** | **UK, Orkney, grooved ware (Holm of papa, Quoyness)** | [22,23] | UK | 5800 | 7800 | 500 | 1 | 6 | 27 |  |  | 34 | 0.18 |
| **20** | **Hulbjerg** | [24,25] | Denmark | 6000 | 8000 | 700 | 2 | 15 | 36 |  |  | 53 | 0.29 |
| **21** | **Kněževes[[6]](#footnote-7)** | [26,27] | Czech republic | 7400 | 9400 | 3200 | 0 | 2 | 6 | 8 |  | 16 | 0.58 |
| **22** | **Germany, Tauber valley, corded ware (Lauda-Konigshofen, Lauda-Konigshofen ortsteil Gerlachsheim, Grünsfeld-Krensheim, Tauberbischofsheim-Dittigheim, Tauberbischofsheim-Impfingen, Tauberbischofsheim-Burgweg, Tauberbischofsheim-Gartnerei Scheuermann, Tauberbischofsheim-Kirchelberg, Werbach)[[7]](#footnote-8)** | [28,29] | Germany | 7400 | 9400 | 2700 | 52 | 82 | 83 |  | 8 | 225 | 0.51 |
| **23** | **Les cres** | [30,31] | France | 7500 | 9500 | 1700 | 4 | 18 | 27 |  |  | 49 | 0.4 |
| **24** | **Monéteau Sur Macherin** | [32] | France | 7100 | 9100 | 1300 |  |  | 11 | 9 | 3 | 23 | 0.44 |
| **25** | **Montou** | [30,33] | France | 7400 | 9400 | 900 | 3 | 2 | 5 |  |  | 10 | 0.29 |
| **26** | **Mulhouse-Est (Rixheim)** | [34] | France | 7400 | 9400 | 150 |  |  | 17 | 9 |  | 26 | 0.32 |
| **27** | **Hungary, Sopot-Bickske culture (Nemesvàmos-Balàka, Bicskse-Galagonyas)** | [15] | Hungary | 7600 | 9600 | 450 | 0 | 3 | 10 |  |  | 13 | 0.23 |
| **28** | **Niedermerz 3** | [34-36] | Germany | 7400 | 9400 | 150 | 0 | 13 | 17 |  |  | 30 | 0.43 |
| **29** | **Pari-Altacker** | [15] | Hungary | 7600 | 9600 | 900 | 0 | 3 | 8 |  |  | 11 | 0.27 |
| **30** | **Passy** | [37-39] | France | 7100 | 9100 | 700 | 5 | 8 | 16 |  |  | 29 | 0.33 |
| **31** | **Point of Cott** | [22,40] | UK | 5800 | 7800 | 500 | 6 | 2 | 5 |  |  | 13 | 0.29 |
| **32** | **Quanterness** | [22] | UK | 5800 | 7800 | 500 | 10 | 62 | 85 |  |  | 157 | 0.42 |
| **33** | **Germany, Mittelelbe-Saale, LBK (Ahlsdorf, Arnstadt, Bad Sulza, Bebertal, Bischleben, Bornstedt, Brachwitz, Ditfurt, Egeln, Eisleben, Erfurt, Grosskorbetha, Grossörner, Grossschwabhausen, Hausneindorf, Korbetha, Köthen, Lebendorf, Leuna-Daspig, Naumburg, Quedlinburg, Westdorf)** | [41] | Germany | 7400 | 9400 | 150 | 1 | 9 | 37 |  |  | 47 | 0.2 |
| **34** | **Germany, Mittelelbe-Saale, stroke-ornamented ware (Röblingen am see, Rothenschirmbach, Seehausen, Spergau, Taubach, Wengelsdorf, Wolmirstedt, Wulfen, Zörbig)** | [41] | Germany | 7400 | 9400 | 800 | 3 | 4 | 8 |  |  | 15 | 0.33 |
| **35** | **Schipluiden** | [42] | Netherlands | 6300 | 8300 | 600 | 2 | 2 | 11 |  |  | 15 | 0.15 |
| **36** | **Schweizersbild** | [43] | Switzerland | 7300 | 9300 | 1300 | 12 | 6 | 12 | 1 |  | 31 | 0.35 |
| **37** | **Sclaigneaux** | [44] | Belgium | 7300 | 9300 | 2200 |  |  | 45 | 13 |  | 58 | 0.23 |
| **38** | **Sengkofen** | [45] | Germany | 7400 | 9400 | 150 | 2 | 4 | 19 |  |  | 25 | 0.17 |
| **39** | **Souffelweyersheim/Hoenheim[[8]](#footnote-9)** | [34] | France | 7400 | 9400 | 150 |  |  | 20 | 5 |  | 25 | 0.21 |
| **40** | **Netherlands, Swifterbant and Urk** | [1,46] | Netherlands | 6300 | 8300 | 200 | 2.8 | 12.56 | 53.64 |  |  | 69 | 0.19 |
| **41** | **Trou Amiault** | [47] | France | 6900 | 8900 | 1250 | 4 | 6 | 7 |  |  | 17 | 0.46 |
| **42** | **Velké Přílepy[[9]](#footnote-10)** | [48] | Czech republic | 7400 | 9400 | 2700 | 1 | 1 | 9 |  | 1 | 12 | 0.18 |
| **43** | **Wayland's Smithy I[[10]](#footnote-11)** | [49,50] | UK | 5900 | 7900 | 150 |  |  | 13 | 1 |  | 14 | 0.11 |
| **44** | **West Kennet** | [51] | UK | 5900 | 7900 | 150 | 6 | 3 | 27 |  |  | 36 | 0.1 |
| **45** | **Wittmar** | [34] | Germany | 7400 | 9400 | 150 | 1 | 5 | 10 |  |  | 16 | 0.33 |
| **46** | **Aisne_serie** | [6,52] | France | 7100 | 9100 | 0 | 10 | 15 | 25 |  |  | 50 | 0.375 |
| **47** | **Aiterhofen_Ődmühle** | [6,45] | Germany | 7400 | 9400 | 150 | 5.4 | 21.43 | 115.17 |  |  | 142 | 0.1569 |
| **48** | **Aven_de_la_Boucle** | [6,53,54] | France | 7500 | 9500 | 1700 | 3.2 | 7.8 | 49 |  |  | 60 | 0.1373 |
| **49** | **Baden_Württemberg** | [6,55] | Germany | 7400 | 9400 | 150 | 7.2 | 12.8 | 11 |  |  | 31 | 0.5378 |
| **50** | **Baume_Bourbon 2** | [6,56,57] | France | 7500 | 9500 | 800 | 1.75 | 3.91 | 9.33 |  |  | 15 | 0.2953 |
| **51** | **Belleville** | [6,58,59] | France | 7200 | 9200 | 2650 | 9.8 | 35.65 | 95.55 |  |  | 141 | 0.2717 |
| **52** | **Breuil_en_Vexin** | [6,59,60] | France | 7100 | 9100 | 2000 | 28 | 20 | 40 |  |  | 88 | 0.3333 |
| **53** | **Brochtorff circle** | [6,61-63] | Malta | 7900 | 9900 | 2000 | 2 | 15 | 55 |  |  | 72 | 0.2142 |
| **54** | **Bruchstedt** | [6,41] | Germany | 7400 | 9400 | 150 | 8.2 | 18.8 | 34 |  |  | 61 | 0.356 |
| **55** | **Cala_Colombo** | [6] | Italy | 7900 | 9900 | 2650 | 4 | 6.25 | 13.75 |  |  | 24 | 0.3125 |
| **56** | **Calle_Sant_Pau** | [6,64] | Spain | 7500 | 9500 | 1750 | 9 | 10 | 7 |  |  | 26 | 0.5882 |
| **57** | **Cauna_de_Belesta 7** | [6,65,66] | France | 7400 | 9400 | 900 | 6 | 11 | 15 |  |  | 32 | 0.423 |
| **58** | **Central_Portugal** | [6,67] | Portugal | 7200 | 9200 | 3100 | 11 | 16 | 103 |  |  | 130 | 0.1344 |
| **59** | **Chamblandes** | [6,68-70] | Switzerland | 7250 | 9250 | 1250 | 25 | 24 | 67 |  |  | 116 | 0.2637 |
| **60** | **Cova_de_Avellaner** | [6,71,72] | Spain | 7500 | 9500 | 800 | 4 | 4 | 11 |  |  | 19 | 0.2666 |
| **61** | **Dedeleben** | [6,73,74] | Germany | 7400 | 9400 | 2450 | 2.6 | 9.42 | 11 |  |  | 23 | 0.4613 |
| **62** | **Derenburg** | [6,73,74] | Germany | 7400 | 9400 | 2450 | 10.8 | 17.22 | 32 |  |  | 60 | 0.3498 |
| **63** | **Diconche** | [6,75] | France | 7000 | 9000 | 2600 | 1 | 2 | 24 |  |  | 27 | 0.0769 |
| **64** | **Eybral** | [6,76,77] | France | 7000 | 9000 | 1900 | 11 | 14 | 50 |  |  | 75 | 0.2187 |
| **65** | **Fontenay-le-Marmion** | [6,78,79] | France | 7000 | 9000 | 1000 | 9.33 | 16.67 | 36 |  |  | 62 | 0.3164 |
| **66** | **Gours_aux_Lions 2** | [6,80-82] | France | 7100 | 9100 | 2000 | 11 | 11 | 32 |  |  | 54 | 0.2558 |
| **67** | **Grossbrembach** | [6,41,83] | Germany | 7400 | 9400 | 3400 | 18.5 | 28.23 | 58.33 |  |  | 105 | 0.3261 |
| **68** | **Hazleton_North** | [6,84,85] | England | 5800 | 7800 | 150 | 3 | 16 | 21 |  |  | 40 | 0.4324 |
| **69** | **Heidelsheim** | [6,86] | Germany | 7400 | 9400 | 1500 | 4.3 | 9.8 | 6.9 |  |  | 21 | 0.5868 |
| **70** | **La_Clape_8** | [6,87] | France | 7500 | 9500 | 3200 | 7 | 14 | 8 |  |  | 29 | 0.6363 |
| **71** | **Laris Goguet** | [6,88] | France | 7100 | 9100 | 2000 | 16 | 16 | 78 |  |  | 110 | 0.1702 |
| **72** | **Lenzburg** | [6,89] | Switzerland | 6500 | 8500 | 500 | 11 | 25.87 | 39.12 |  |  | 76 | 0.398 |
| **73** | **Les Mournouards 2** | [6,60,90] | France | 7100 | 9100 | 2000 | 9.23 | 19.77 | 31 |  |  | 60 | 0.3894 |
| **74** | **Loisy_en_Brie** | [6,91] | France | 7100 | 9100 | 2000 | 19 | 31 | 114 |  |  | 164 | 0.2137 |
| **75** | **Maillets** | [6,92,93] | France | 7100 | 9100 | 2000 | 5 | 4.5 | 33 |  |  | 42.5 | 0.12 |
| **76** | **Malesherbes_Orville “les Fiefs”** | [6,94] | France | 7100 | 9100 | 700 | 1 | 5.5 | 17.5 |  |  | 24 | 0.2391 |
| **77** | **Monte_Canelas 1** | [6] | Portugal | 7000 | 9000 | 1880 | 25 | 25 | 97 |  |  | 147 | 0.2049 |
| **78** | **Montigny-Esbly** | [6,60,95] | France | 7100 | 9100 | 2000 | 6 | 26 | 79 |  |  | 111 | 0.2476 |
| **79** | **Moragy_B_1** | [6,15] | Hungary | 7600 | 9600 | 900 | 16.2 | 25.8 | 39 |  |  | 81 | 0.3981 |
| **80** | **Niederbosa** | [6,41,96] | Germany | 7400 | 9400 | 2000 | 14.4 | 28.6 | 50 |  |  | 93 | 0.3638 |
| **81** | **Nitra** | [6,97] | Slovakia | 7600 | 9600 | 350 | 12 | 13 | 47 |  |  | 72 | 0.2166 |
| **82** | **Nordhausen** | [6,41,83,98] | Germany | 7400 | 9400 | 2400 | 3 | 23 | 24 |  |  | 50 | 0.4893 |
| **83** | **Octrois, Ensisheim** | [6,99] | France | 7300 | 9300 | 50 | 2 | 6 | 33 |  |  | 41 | 0.1538 |
| **84** | **Paradis** | [6,100] | France | 7100 | 9100 | 2000 | 2 | 7.5 | 7.5 |  |  | 17 | 0.5 |
| **85** | **Pech_1** | [6,101] | France | 7200 | 9200 | 2900 | 7 | 7 | 28 |  |  | 42 | 0.2 |
| **86** | **Pierre_Folle** | [6,102,103] | France | 7000 | 9000 | 2700 | 5 | 11 | 24 |  |  | 40 | 0.3142 |
| **87** | **Pontcharaud_2** | [6,104,105] | France | 7000 | 9000 | 800 | 21 | 22.42 | 54.58 |  |  | 98 | 0.2911 |
| **88** | **Reaudins - Balloy** | [6,37,38,106-108] | France | 7100 | 9100 | 700 | 8 | 7 | 24 |  |  | 39 | 0.2258 |
| **89** | **Rutzing_Haid Rutzing** | [6] | Austria | 7400 | 9400 | 150 | 0 | 2 | 9 |  |  | 11 | 0.1818 |
| **90** | **Schonstedt** | [6,41,73] | Germany | 7400 | 9400 | 2000 | 17.1 | 18.9 | 28 |  |  | 64 | 0.4029 |
| **91** | **Sondershausen** | [6,41] | Germany | 7400 | 9400 | 150 | 4.2 | 10.8 | 32 |  |  | 47 | 0.2523 |
| **92** | **Stuttgart** | [6,109] | Germany | 7400 | 9400 | 150 | 3.6 | 20.39 | 58 |  |  | 82 | 0.2601 |
| **93** | **Trebur** | [6,110] | Germany | 7400 | 9400 | 550 | 6.17 | 17.39 | 81.44 |  |  | 105 | 0.1759 |
| **94** | **Vedrovice** | [6,111] | Czech Republic | 7500 | 9500 | 100 | 15 | 12 | 77 |  |  | 104 | 0.1348 |
| **95** | **Vikletice** | [6,112] | Czech Republic | 7400 | 9400 | 2700 | 22.5 | 28.5 | 90 |  |  | 141 | 0.2404 |
| **96** | **Villaine** | [6,113,114] | France | 6900 | 8900 | 1800 | 21 | 17.33 | 96.67 |  |  | 135 | 0.152 |
| **97** | **Villany-kovesd** | [6,115] | Hungary | 7800 | 9800 | 1100 | 5 | 5 | 14 |  |  | 24 | 0.2631 |
| **98** | **Wandersleben** | [6,116] | Germany | 7400 | 9400 | 150 | 38 | 60 | 118 |  |  | 216 | 0.337 |
| **99** | **Zengovarkony** | [6,15,117] | Hungary | 7800 | 9800 | 1100 | 3 | 5 | 56 |  |  | 64 | 0.0819 |
| **100** | **Totenhütte, Benzingerode[[11]](#footnote-12)** | [118] | Germany neo | 7400 | 9400 | 2400 | 5 | 8 | 22 | 9 | 2 | 46 | 0.35 |
| **101** | **Sammelserie** | [6] | Germany | 7400 | 9400 | 150 | 6.6 | 15.4 | 59 |  |  | 81 | 0.2069 |

# REFERENCES

1. Smits L, Van der Plicht H (2009) Mesolithic and Neolithic human remains in the Netherlands: physical anthropological and stable isotope investigations. Journal of Archaeology in the Low Countries 1: 55-85.

2. Péquart M, Péquart ST, Vallois HV (1954) Hoëdic, deuxième station-nécropole du Mesolithique côtier armoricain. Antwerp: De Sikkel.

3. Schulting RJ (1999) Nouvelles dates AMS à Téviec et Hoëdic (Quiberon, Morbihan). Bulletin de la Société Préhistorique française 96: 203-207.

4. Schulting RJ, Richards MP (2001) Dating women and becoming farmers: new palaeodietary and AMS dating evidence from the Breton Mesolithic cemeteries of Téviec and Hoëdic. Journal of Anthropological Archaeology 20: 314-344.

5. Péquart M, Péquart ST, Boule M, Vallois HV (1937) Téviec: station-nécropole mésolithique du Morbihan. .

6. Bocquet-Appel J (2002) Paleoanthropological Traces of a Neolithic Demographic Transition. Current Anthropology 43: 637-650.

7. Jackes M, Roksandić M, Meiklejohn C (2001) Demography of the Ðerdap Mesolithic-Neolithic transition. In: Bonsall C, Boroneant V, Radovanovic I, editors. The Iron gates in prehistory. New perspectives. Oxford: Archaeopress. pp. 77-88.

8. Ferembach D (1974) Le gisement Néolithique de Moita do Sebastião, Muge, Portugal. Lisbon: Direcção-Geral dos Assuntos culturais.

9. Jackes M (2009) Teeth and the past in Portugal: pathology and the Mesolithic-Neolithic transition. In: Koppe T, Meyer G, Alt KW, editors. Comparative dental morphology. 14th international symposium on dental morphology, Greifswald, August 2008. Basel: Karger. pp. 167-172.

10. Meiklejohn C, Wymann JM, Jacobs K, Jackes M (1997) Issues in the archaeological demography of the agricultural transition in Western and Northern Europe: a view from the Mesolithic. In: Paine PR, editors. Integrating archaeological demography. Carbondale, pp. 311-326.

11. Albrethsen SE, Petersen EB (1976) Excavation of a Mesolithic cemetery at Vedbaeck, Denmark. Acta Archaeologica 47: 1-28.

12. Bayliss A, Benson D, Galer D, Humphrey L, McFadyen L, Whittle A (2007) One thing after another: the date of the Ascott-under-Wychwood long barrow. Cambridge Archaeological Journal 17: 29-44.

13. Galer D (2007) The human remains. In: Benson D, Whittle A, editors. Building memories: the Neolithic Cotswold long barrow at Ascott-under-Wychwood, Oxfordshire. Oxford: Oxbow Books. pp. 189-218.

14. Kalicz N (1985) Kőkori falu Aszódon (Neolithisches Dorf in Aszód). Aszód: Petőfi Múzeum.

15. Zoffmann Z (1999) Anthropological data of the Transdanubian prehistoric populations in the Neolithic, the Copper, the Bronze and the Iron ages. Savaria 24: 33-49.

16. Neustupny E, Smrž Z (1989) Cachovice - pohrebiste kultury se snurovou keramikou a zvoncovitych poharu Cachovice: une nécropole des cultures à céramique cordée et campaniforme. Památky Archeologické 80: 282-283.

17. Chamberlain AT, Williams JP (2001) A gazetteer of English caves, fissures and rock shelters containing human remains, revised version June 2001. Capra 1.

18. Desideri J, Eades S (2002) La question du "peuplement" campaniforme en Suisse Occidentale. Etat de la question et apports de la morphologie dentaire et cranienne. Bulletins et Mémoires de la Société Préhistorique d'Anthropologie de Paris 14: 345-362.

19. Honegger M, Desideri J (2003) Archéologie et anthropologie de la nécropole du Néolithique moyen de Barmaz (Vallais, Suisse). In: Chambon P, Leclerc J, editors. Les pratiques funéraires néolithiques avant 3500 avant J-C. en France et dans les régions limitrophes. Table Ronde SPF, Saint-Germain-en-Laye, 15-17 Juin 2001. Paris: Société Préhistorique Française, pp. 219-230.

20. Baudais D, Kramar C (1990) La nécropole Néolithique de Corseaux "en Seyton". Lausanne: Bibliothèque historique vaudoise.

21. Wysocki M, Bayliss A, Whittle A (2007) Serious mortality: the date of the Fussell's lodge long barrow. Cambridge Archaeological Journal 17: 65-84.

22. Reilly S (2003) Processing the dead in Neolithic Orkney. Oxford Journal of Archaeology 22: 133-154.

23. Ritchie A (2009) On the fringe of Neolithic Europe: excavation of a chambered cairn on the Holm of Papa Westray, Orkney. Edinburgh: Society of Antiquaries of Scotland.

24. Bennike P (1985) Palaeopathology of Danish skeletons: a comparative study of demography, disease and injury. Copenhagen: Akademisk Forlag.

25. Fischer A, Olsen J, Richards M, Heinemeier J, Sveinbjörnsdóttir AE, Bennike P (2007) Coast-inland mobility and diet in the Danish Mesolithic and Neolithic: evidence from stable isotope values of humans and dogs. Journal of Archaeological Science 34: 2125-2150.

26. Desideri J, Besse M (2010) Swiss Bell Beaker population dynamics: eastern or southern influences? Archaeological and Anthropological Sciences 2: 157-173.

27. Kytlicová O (1956) Nécropole de la civilisation à vases campaniformes à Kneževes. Archeologické Rozhledy 8: 358-359.

28. Menninger M (2008) Die schnurkeramischen Bestattungen von Lauda-Königshofen. Steinzeitliche Hirtennomaden im Taubertal? Eberhard-Karls-Universität Tübingen.

29. Dresely V (2004) Schnurkeramik und schnurkeramiker im Taubertal. Stuttgart: K. Theiss.

30. Herrscher E, Le Bras-Goude G (2010) Southern French Neolithic populations: isotopic evidence for regional specificities in environment and diet. American Journal of Physical Anthropology 141: 259-272.

31. Le Bras-Goude G, Schmitt A, Loison G (2009) Comportements alimentaires, aspects biologiques et sociaux au Néolithique: le cas du Crès. Compte Rendu Palevol 8: 79-91.

32. Augereau A, Chambon P (2005) Les occupations Néolithiques de Monéteau "Sur Macherin" (Yonne): Données préliminaires. Revue Archéologique de l'Est 54: 51-70.

33. Valentin F, Donat R, Claustre F (2003) La gestion de l'espace sépulcral Néolithique moyen de la grotte de Montou (Pyrénées-Orientales): un essai d'interprétation. Bulletin de la Société Préhistorique française 23: 301-314.

34. Peschel C (1992) Regel und Ausnahme: Linearbandkeramische bestattungssitten in Deutschland und angrenzenden gebieten, under besonderer berücksichtigung der sonderbestattungen. Rahden: Verlag M Leidorf.

35. Dohrn-Ihmig M (1983) Das bandkeramische gräberfeld von Aldenhoven-Niedermerz, Kreis Düren. In: Bauchhenss G, editors. Archäologie in den Rheinischen Lössbörden: Beitrage zur Siedlungsgeschichte im Rheinland. Bonn: Habelt.

36. Dolukhanov P, Shukurov A, Gronenborn D, Sokoloff D, Timofeev V, Zaitseva G (2005) The chronology of Neolithic dispersal in Central and Eastern Europe. Journal of Archaeological Science 32: 1441-1458.

37. Dubouloz J (2003) Datation absolue du premier Néolithique du Bassin parisien: complément et relecture des données RRPB et VSG. Bulletin de la Société Préhistorique française 100: 671-689.

38. Duhamel P, Mordant D (1997) Les necropolis monumentales Cerny du basin Seine-Yonne. In: Constantin C, Simonin D, editors. La culture de Cerny: nouvelle économie, nouvelle société au Néolithique: actes du colloque international de Nemours, 9-10-11 mai 1994. Nemours: APRAIF. pp. 481-488.

39. Duhamel P (1997) La nécropole monumentale de Cerny de Passy (Yonne): description d'ensemble et problèmes d'interprétation. In: Constantin C, Mordant D, Simonin D, editors. La culture de Cerny: nouvelle économie, nouvelle société au Néolithique. Actes du colloque international de Nemours, 9-10-11 Mai 1994. Nemours: APRAIF. pp. 397-448.

40. Barber J (1997) The excavation of a stalled cairn at the Point of Cott, Westray, Orkney. Edinburgh: Scottish Trust for Archaeological Research.

41. Bach A (1978) Neolithische Populationen im Mittelelbe-Saale-Gebiet: zur Anthropologie des Neolithikums unter besonderer Berücksichtigung der Bandkeramiker. Weimar: Museum für Ur-und Frühgeschichte Thüringens.

42. Smits E, Louwe Kooijmans LP (2006) Graves and human remains. Analecta Praehistorica 37-38: 91-112.

43. Höneisen M, Peyer S (1994) Schweizersbild-ein jägerlager der Späteiszeit. Beitrage und Dokumente zur Ausgrabung vor 100 Jahren. Schaffhausen: Kantousarchäologie.

44. De Paepe M, Pollet C (2007) "Numerous and tall": a revision of the late Neolithic human remains found in a collective burial at Sclaigneaux (prov. Namur, Belgium). Notae Prehistoricae 27: 163-168.

45. Nieszery N (1995) Linearbandkeramische gräberfelder in Bayern. Espelkamp: Verlag Marie L. Leidorf.

46. Constandse-Westermann TS, Meiklejohn C (1979) The human remains from Swifterbant. Helinium 19: 235-260.

47. de Souris L (2007) Occupations funéraires des grottes Néolithiques en Poitou-Charentes: l'exemple du Trou Amiault (La Rochette, Charente, France). In: Besse M, editors. Sociétés Néolithiques: des faits archéologiques aux fonctionnnements socio-économiques. Actes du 27ème colloque international sur le Néolithique. Neuchâtel 1 et 2 Octobre 2005. Lausanne: Cahiers d'archéologie romande.

48. Smejtek L, Vojtechovská I (1999) Archäologische Ausgrabungen in Velké Prílepy, Bez Prag-West. Belege der Besiedlung vom Ende des Äneolithikums und Bronzeit. In: Bátora J, Pejka J, editors. Aktuelle Probleme der Frühbronzezeit in Böhmen und Mähren und der Slowakei. Nitra. pp. 21-32.

49. Smith M, Brickley M (2009) People of the long barrows: life, death and burial in the earlier Neolithic. Stroud: History Press.

50. Whittle A, Bayliss A, Wysocki M (2007) Once in a lifetime: the date of the Wayland's Smithy long barrow. Cambridge Archaeological Journal 17: 103-121.

51. Bayliss A, Whittle A, Wysocki M (2007) Talking about my generation: the date of West Kennet long barrow. Cambridge Archaeological Journal 17: 85-101.

52. Constantin C, Farruggia JP, Bonnardin S, Guichard Y, Sidéra I (2003) Les tombes rubanées de la vallée de l'Aisne. Présentation. In: Chambon P, Leclerc J, editors. Les pratiques funéraires Néolithiques avant 3500 avant J.C. en France et dans les régions limitrophes. Paris: Société Préhistorique Française. pp. 55-63.

53. Duday H (1980) Les rites funéraires en Languedoc au cours du troisième millénaire. In: Guilaine J, editors. Le groupe de Véraza et la fin des temps Néolithiques dans le sud de la France et la Catalogne. Paris: Editions du CNRS. pp. 273-282.

54. Duday H (1987) Organisation et fonctionnement d'une sépulture collective Néolithique l'Aven de la boucle à Courconne (Gard). In: Masset C, editors. Anthropologie physique et archéologie: méthodes d'étude des sépultures: actes du colloque de Toulouse 4, 5 et 6 Novembre 1982. Paris: Editions de la CNRS. pp. 89-97.

55. Orschiedt J (1997) Sépultures rubanées en habitat dans le Baden-Württemberg. Etudes archéologiques et anthropologiques. Actes du 22ème colloque interrégional sur le Néolithique, Strasbourg 27-29 Octobre 1995. Cahiers de l'association pour la promotion de la recherche archéologique en Alsace : 57-63.

56. Beyneix A (1997) Les sépultures cardiales et épicardiales de France méridionales. Bulletin de la Société Préhistorique française 94: 191-197.

57. Coste A, Duday H, Roudil JL, Gutherz X (1983) Les sépultures de la baume Bourbon à Cabrières (Gard). In: Guilaine J, editors. Premières communautés paysannes en Méditerrannée occidentale. Colloque international du CNRS, 26-29 Novembre, Montpellier. Paris: Editions CNRS. pp. 531-535.

58. Baudouin M (1911) La sépulture Néolithique de Belleville à Vendrest (Seine et Marne): fouille et restauration. Paris: Société Préhistorique Française.

59. Gatto E (2007) La crémation parmi les pratiques funéraires du Néolithique récent-final en France. Méthodes d'étude et analyse de sites. Bulletins et Mémoires de la Société Préhistorique d'Anthropologie de Paris 19: 195-220.

60. Chambon P, Salanova L (1996) Chronologie des sépultures du IIIème millénaire dans le bassin de la Seine. Bulletin de la Société Préhistorique française 93: 103-118.

61. Richards MP, Hedges REM, Walton I, Stoddart S, Malone C (2001) Neolithic diet at the Brochtorff circle, Malta. European Journal of Archaeology 4: 253-262.

62. Malone C, Stoddart S, Bonanno A, Gouder T, Trump D, et al (1995) Mortuary ritual of 4th millennium BC Malta: the Zebbug period chambered tomb from the Brochtorff Circle at Xaghra (Gozo). Proceedings of the Prehistoric Society 61: 303-345.

63. Malone C, Stoddart S, Trump D, Bonanno A, Pace A (2009) Mortuary ritual in prehistoric Malta. The Brochtorff Circle excavations (1987-1994). Cambridge: McDonald Institute.

64. Anfruns J, Majo T, Oms JI (1991) Estudio preliminar de los restos humanos neolíticos procedentes del yacimiento de la calle Sant Pau (Barcelona). In: Botella MC, editors. Nuevas perspectivas en antropología. Congreso español de antropología biologica (7). Granada: Diputación provincial de Granada.

65. Cauwe N (1999) A propos des sépultures collectives dans le groupe de Montbolo. In: Bernabeu Auban J, Orozco Kîhler T, editors. II congrés del Neolitic a la peninsula Ibèrica, Universitat de València 7-9 d'Abril. Valencia: Universitat de Valencia. pp. 453-459.

66. Claustre F, Zammit J, Blaize Y (1993) La Caune de Bélesta: une tombe collective il y a 6000 ans. Toulouse: Centre d'Anthropologie des Sociétés Rurales, CNRS.

67. Silva AM (1996) Paleobiology of the population inhumated in the hipogeum of Monte Canelas I (Alcalar), Portugal. In: Facchini F, editors. Proceedings of the XIII Congress of the International Union of Prehistoric and Protohistoric Sciences. Forli, Italy: ABACO. pp. 137-146.

68. Moinat P, Simon C (1986) Nécropole de Chamblandes-Pully, Nouvelles observations. Jahrbuch der Schweizerischen Gesellschaft für Ur-und Frühgeschichte 69: 39-53.

69. Moinat P (1994) Cistes néolithiques et incinération du Bronze final à Pully VC-Chamblandes. Jahrbuch der Schweizerischen gesellschaft für Ur-und Frühgeschichte 77: 123-126.

70. Skeates R (1993) Mediterrannean coral: its use and exchange in and around the Alpine region during the later neolithic and copper age. Oxford Journal of Archaeology 12: 281-292.

71. Manen C, Sabatier P (2003) Chronique radiocarbone de la néolithisation en Méditerrannée nord-occidentale. Bulletin de la Société Préhistorique française 100: 479-504.

72. Mercadal Fernández O, Defaus I, Congost JM, Alacambra I, Sánchez PJ, et al (1990) L'anàlisi anthropologic. In: Boch I, Lloret A, Tarrús I, Galter J, editors. La cova sepulchral del Neolític antic de l'Avellaner (Cogolls, Les Planes, La Garotxa). Girona: Centre d'Investigacons Arqueològiques de Girona.

73. Bach A (1981) Skelettreste aus zwei kollektivgräbern der Bernburger Kultur (Derenburg, Kr. Wernigrode, und Dedeleben, Kr. Halberstadt). Jahresschrift für Mitteldeutsche Vorgeschichte 63: 67-74.

74. Behrens H (1981) Radiocarbon-daten für das Neolithikum des Mittelelbe-Saale-Gebiets. Jahresschrift für mitteldeutsche Vorgeschichte 63: 189-193.

75. Semlier P (1999) Le matérial anthropologique. In: Burnez C, Fouéré P, editors. Les enceintes Néolithiques de Diconches à Saintes (Charente-Maritime): une périodisation de l'Artenac. Chauvigny, Association des Publications chauvinoises (Mémoires, XV) et Paris, Société Préhistorique française (Mémoires, XXV).

76. Ben-Ncer A (1991) La sépulture collective d'Eybral (Coux-et-Bigaroque, Dordogne): étude anthropologique. Université de Bordeaux 1, France.

77. Rigaud JP (1976) Aquitaine. Gallia Préhistoire 19: 523-546.

78. Dastugue J, Torre S (1972) Le cairn et le crématoire Néolithiques de la Hoguette à Fontenay-le-Marmion (Calvados). Gallia Préhistoire 15: 187-197.

79. Dastugue J, Torre S, Buchet L (1973) Néolithiques de Basse-Normandie. Le deuxième tumulus de Fontenay-le-Marmion. L'anthropologie 77: 579-620.

80. Baron R, Demetz JL, Monmignaut C (1967) Les sépultures de Marolles-sur-Seine (Seine et Marne): les hommes de la sépulture II. Gallia Préhistoire 10: 140-155.

81. Mordant D (1965) Le site des Gours-aux-lions, à Marolles-sur-Seine (Seine et Marne). Bulletin de la Société Préhistorique française 62: 715-716.

82. Masset C, Mordant C, Mordant D (1967) Les sépultures collectives de Marolles-sur-Seine (Seine et Marne). Gallia Préhistoire 10: 74-136.

83. Ullrich H (1972) Das aunjetitzer gräberfeld von Grossbrembach. Weimar: Hermann Boehlaus.

84. Meadows J, Barclay A, Bayliss A (2007) A short passage of time: the dating of the Hazleton long cairn revisited. Cambridge Archaeological Journal 17: 45-64.

85. Rogers J (1990) The human skeletal material. In: Saville A, editors. Hazleton North: the excavation of a Neolithic long cairn of the Cotswold-Severn group. London: English Heritage. pp. 182-198.

86. Lichardus J, Guilaine J (1986) Le ritual funéraire de la culture de Michelsberg dans la region du Rhin supérieur et moyen. In: Demoule P, editors. Le Néolithique de la France. .

87. Guilaine J (1972) La nécropole mégalithique de la Clape (Laroque-de-Fa, Aude). Carcassonne: Laboratoire d'Anthropologie de l'Université de Bordeaux 1.

88. Bendezu-Sarmiento J (1999) Le "Laris-Goguet" à Feigneux (Oise), une grotte sépulcrale de la fin du Néolithique. De nouvelles données à partir d'une étude, archéologique et anthropologique, effectuée sur les sujets immatures. Revue Archéologique de Picardie 1-2: 63-82.

89. Scheffrahn W (1967) Palaeodemographische beobachtungen an den Neolithikern von Lenzburg. Germania 45: 34-42.

90. Leroi-Gourhan A, Bailloud G, Brezillon M, Monmignaut C (1962) L'hypogée II des Mournouards (Mesnil-sur-Oger, Marne). Gallia Préhistoire 5: 23-133.

91. Bocquet-Appel JP (1994) L'hypogée néolithique de Loisy-en-Brie (Marne), lieu-dit Les Gouttes d'or: l'interprétation démographique. Préhistoire et Protohistoire en Champagne Ardenne 18: 55-60.

92. Baumann F, Tarràte J (1979) La sépulture des Maillets à Germigny-L'évêque (Seine et Marne): les fouilles et la structure: Interprétation. Gallia Préhistoire 22: 143-153.

93. Patte E (1979) La sépulture des Maillets à Germigny-L'évêque (Seine et Marne): étude anthropologique. Gallia Préhistoire 22: 179-200.

94. Simonin D, Bach S, Richard G, Vintrou J (1997) Les sépultures sous dalles de type Malesherbes et la nécropole d'Orville. In: Constantin C, Mordant D, editors. La culture de Cerny: nouvelle économie, nouvelle société au Néolithique, actes du colloque international de Nemours, 9-10-11 Mai 1994. Nemours: APRAIF. pp. 341-379.

95. Masset C (1975) Problèmes de démographie préhistorique. Université de Paris 1.

96. Bach A (1979) Zur Anthropologie des Neolithikums im Mittelelbe-Saale Gebiet unter besonderer berücksichtigung der Bandkeramiker. Weimar: Museum für Ur-und Frühgeschichte Thüringens.

97. Pavúk J (1972) Neolithische gräberfeld in Nitra. Slovenská archeológia 20.

98. Feustel R, Ullrich H (1965) Totenhütten der Neolithische Walternienburger gruppe. Alt-Thuringen 4: 104-202.

99. Jeunesse C (2012) Pratiques funéraires au Néolithique ancien: sépultures et nécropoles des sociétés danubiennes (5500-4900 avant J.C.). Paris: Editions errance.

100. Girard C (1973) Les vestiges anthropologiques. In: Brezillon M, editors. La sépulture collective du Paradis à Noisy-sur-Ecole (Seine-et-Marne). Paris: U.E.R. d'art et d'archeologie. pp. 20-31.

101. Carrière M, Clottes J (1970) Le dolmen du Pech n° 1 à Alvignac (Lot). Gallia Préhistoire 13: 109-149.

102. Brabant H (1976) Le dolmen Angevin de Pierre-Folle à Thiré (Vendée): étude odontologique. Gallia Préhistoire 19: 51-60.

103. Joussaume R (1976) Le dolmen Angevin de Pierre-Folle à Thiré (Vendée): étude architecturale et archéologique. Gallia Préhistoire 19: 1-37.

104. Gisclon JL (1993) La nécropole néolithique de Pontcharaud, Clermont-Ferrand: rapport anthropologique de synthèse. Bordeaux: Laboratoire d'Anthropologie de l'Université de Bordeaux 1.

105. Loison G (1998) La nécropole de Pontcharaud en Basse-Auvergne. In: Guilaine J, editors. Sépultures d'Occident et génèses des mégalithisimes (9000-3500 avant notre ère). Paris: Editions Errance. pp. 189-206.

106. Chambon P (1997) La nécropole de Balloy "les Réaudins" approche archéo-anthropologique. In: Constantin C, Mordant D, Simonin D, editors. La culture de Cerny: nouvelle économie, nouvelle société au Néolithique. Actes du colloque international de Nemours, 9-10-11 Mai 1994. Nemours: APRAIF. pp. 489-498.

107. Constantin C, Ilett M (1997) Une étappe finale dans le Rubané récent du Bassin parisien. In: Jeunesse C, editors. Le Néolithique danubien et ses marges entre Rhin et Seine, Actes du 22ème colloque international sur le Néolithique, Strasbourg, 27-29 Octobre 1995. Strasbourg: APRAA. pp. 27-29.

108. Mordant D (1997) Le complexe des Réaudins à Balloy: enceinte et nécropole monumentale. In: Constantin C, Simonin D, editors. La culture de Cerny: nouvelle économie, nouvelle société au Néolithique. Actes du colloque international de Nemours, 9-10-11 mai 1994. Nemours: APRAIF. pp. 449-479.

109. Seitz M (1989) Das linearbandkeramische gräberfeld von Stuttgart-Mühlhausen, Viesenhäuser hof. Tübingen university.

110. Spatz H (1999) Das mittelneolithische gräberfeld von Trebur, Kreis Gross-Gerau, Materialien zur Vor-und Frühgeschichte von Hessen. Wiesbaden: Selbstverlag des Landesamtes für Denkmalpflege Hessen.

111. Crubézy E, Murail P, Bruzek J, Jelinek J, Ondrus V, et al (1997) Sample characterization of Danubian cemeteries in Central Europe: the examples of Verdovice (Moravia) and Nitra-Horne Krskany (Slovakia). In: Jeunesse C, editors. Le Néolithique danubien et ses marges entre Rhin et Seine. Actes du 22ème colloque international sur le Néolithique. Strasbourg: APRAA. pp. 9-16.

112. Buchvaldek M, Koutecky D (1970) Vikletice. Ein Schnurkeramische Gräberfeld. Prague: University Karlova.

113. Brabant H (1972) Etude des dents. Gallia Préhistoire 15: 111-128.

114. Cordier G (1972) Etude archéologique. Gallia Préhistoire 15: 31-92.

115. Zoffmann Z (1968) An anthropological study of the Neolithic cemetery at Villánykövesd (Lengyel culture), Hungary. Annales musei de Iano Pannonio Nominati 13: 25-39.

116. Bach A (1986) Einige Befunde an den Skeletten aus den Körpergräbern des linienbandkeramischen Gräberfeldes von Wandersleben, Kr. Gotha. Anthropologie 24: 111-144.

117. Zoffmann Z (1969) Anthropological analysis of the cemetery at Zengövárkony and the Neolithic Lengyel culture in SW Hungary. Annales Musei de Lano Pannonio Nominati 14: 53-73.

118. Berthold B, Alt KW, Bramanti B, Drings S, Kranzbühler J, et al (2008) Die Totenhütte von Benzingerode. Archäologie und Anthropologie. Halle: Landesamt für Denkmalpflege und Archäologie Sachsen-Anhalt - Landesmuseum für Vorgeschichte.

1. Original reference mentions 9 adults, 2 neonates, 1 ind. 3-5 yrs. and 2 "children". More recent analysis [4] has assigned 3 ind. to a 0-2 age category and 2 ind. to a 2-12 age category. [↑](#footnote-ref-2)
2. Original reference mentions 14 adults, 1 adolescent (14-16), 3 ind. 0-4, 3 "children", 1 ind. 3-5, 1 "very small child". The most recent analysis [4] assigned 6 ind. to 0-2 age category, 2 ind. to 2-12 age category and re-assigned adolescent to the adult category. [↑](#footnote-ref-3)
3. All cemeteries Bocquet-Appel used in [6] were included for comparative purposes, including a small number from outside Europe. [↑](#footnote-ref-4)
4. Skeletal information from nine graves omitted because no age-structure information was recorded (remains destroyed or too fragmented). Original reference [16] page 382 mentions 39 adults, 2 ind. 0-4, 8 ind. 5-19 and 2 “children”. [↑](#footnote-ref-5)
5. Original reference mentions 42 adults, 4 ind. 0-4, 9 ind. 5-19, 1 ind. 3-5 and 14 “immatures” (sic.). [↑](#footnote-ref-6)
6. Original reference mentions 6 adults, 2 ind. 5-19 and 8 “children”. [↑](#footnote-ref-7)
7. 4 Impfingen skeletons damaged post-excavation. Photographs used to identify 2 as adults (added to “adults”) and 2 to “subadults”. The remaining 4 undetermined skeletons from Dittigheim were assumed not to be “Infants”. [↑](#footnote-ref-8)
8. Original reference [34] page 127 mentions 20 adults, 4 « infans » (0-14) and 1 « juvenile » (14-19). [↑](#footnote-ref-9)
9. 2 skeletons listed as “adults?” classified as 16-50. [↑](#footnote-ref-10)
10. 1 child with “small unfused epiphyses” classified 0-15. [↑](#footnote-ref-11)
11. Two unknown individuals were too fragmented to reliable assess age. [↑](#footnote-ref-12)
